# Emulsion Gels Stabilized Using Sweet Almond Expeller Globulin Modified by Ultrasonication Assisted with Chlorogenic Acid Conjugation: Preparation, Characterization, Stability, and Applications in Curcumin

**DOI:** 10.3390/foods15183213

**Published:** 2026-09-11

**Authors:** Yi Li, Ling Dang, Biao Ma, Yanan Ren, Yajun Zheng

**Affiliations:** 1 Food Science College, Shanxi Normal University, Taiyuan 030092, China; 19035853737@163.com (Y.L.); 19213510834@163.com (B.M.); 13453283830@163.com (Y.R.); 2 School of Health Management, Shanxi Technology and Business University, Taiyuan 030006, China

**Keywords:** sweet almond expeller globulin, emulsion gel, chlorogenic acid conjugation, structural characteristics, textural properties, rheological stability, curcumin bioaccessibility

## Abstract

Almond expeller is high in protein content and produced in large quantities every year; however, its utilization in the food industry remains limited. To provide new strategies to enhance the performance of protein-based emulsion gels and expand the applications of sweet almond expeller globulin (SAEG) in the food industry, emulsion gels were formed using sweet almond expeller globulin modified by ultrasonication and chlorogenic acid conjugation (SAEG-UCA) in this study. The results showed that ultrasonication and chlorogenic acid grafting improved the emulsifying capacity (from 67.51 to 143.65 m^2^/g) and emulsion stability (from 70.37% to 87.18%) of SAEG by increasing its solubility and interface sorption capacity and enhancing the zeta potential within the emulsion (*p* < 0.05). Compared with the SAEG-emulsion gel, the SAEG-UCA-emulsion gel exhibited a more compact and denser gel structure, higher crystallinity (30.71%), bound water content and viscosity, and superior rheological stability (lower loss factor) and elasticity. Furthermore, the SAEG-UCA-emulsion gel showed superior oxidative stability, and higher chewiness (32.35 g), springiness (0.77), hardness (78.63 g), and cohesiveness (0.72) than SAEG-emulsion gel. Additionally, the SAEG-UCA-emulsion gel improved the photostability, encapsulation and loading capacities, and bioaccessibility of curcumin (*p* < 0.05). However, more specific mechanisms should be investigated in future research.

## 1. Introduction

Sweet almond (*Prunus armeniaca*) is widely planted in China, especially in Shanxi Province, Hebei Province, and the Xinjiang Uygur Autonomous Region [1]. Sweet almond milk is an important plant-based non-dairy milk alternative. The total sales volume of almond milk in 2024 was approximately 248,000 tons in China [2]. Almond expeller is a by-product of almond milk production and is primarily used for feed processing. The protein content of sweet almond expeller is 42–61 g/100 g. Globulin accounts for approximately 50.67 g/100 g of sweet almond expeller protein [3]. Although the functionalities and health care activities of almond protein isolates have been widely investigated [4,5,6], data on sweet almond expeller globulin (SAEG) are scarce. SAEG showed superior digestibility (92.63%) and emulsifying properties compared with soy proteins, but its emulsifying capacity was lower than that of Tween 40 and sodium caseinate [7], indicating that it can be used as a natural emulsifier if its emulsifying properties are improved. However, data regarding the improvement in emulsifying properties of SAEG and underlying mechanisms are limited.

Emulsions are widely used in the food industry; however, emulsions are mostly thermodynamically unstable, which limits their widespread applications [8]. By contrast, emulsion gels have adjustable elasticity and better stability relative to emulsions and gels [9]. Emulsion gels are semi-solid or solid materials with both the rheological properties of emulsions and the mechanical properties of gels. Prior studies demonstrated that emulsion gels significantly enhanced the stability and bioavailability of lutein esters, curcumin, astaxanthin and other bioactive compounds [7,8]. In recent decades, the anti-inflammatory, cardiovascular- and cerebrovascular-protective, and anti-cancer effects of curcumin have been demonstrated in numerous clinical trials [10]. However, prior studies found that the bioavailability and aqueous solubility of curcumin were low, and its stability against light, thermal treatment, and alkali and acidic conditions is poor [11,12]. Although some emulsion gels based on proteins (such as egg white and soybean proteins, and zein), or protein-polysaccharide and protein-polyphenols complexes have been used to deliver curcumin to improve its stability and bioavailability [10,13], data regarding curcumin delivery systems based on modified proteins are limited.

More importantly, emulsifiers and gelatinizers are crucial for the formation of a stable emulsion gel. Compared to chemically synthesized emulsifiers and gelatinizers, food-derived proteins and their derivatives are better candidates due to their better biodegradability, biocompatibility, and superior safety [14]. However, most natural proteins exhibit relatively poorer emulsion properties than chemically synthesized emulsifiers such as Span 80, Tween 80, and sodium caseinate [15]. To improve the emulsion gel properties of proteins, glycosylation, polyphenol conjugation, ultrasonication, and high-speed shearing have been employed [5,16,17]. Ultrasonication generates cavitation effects, which can break chemical bonds and expand the structure of proteins, exposing more polar or non-polar groups, enhancing the interfacial adsorption ability of polypeptide chains, and thereby improving their emulsifying properties [18]. Polyphenol conjugation is another effective method for improving the emulsifying properties of proteins. The introduced polyphenolic groups enhanced the binding affinity of the proteins to oil, increasing their interface sorption ability and reducing interfacial tension [19,20]. Chlorogenic acid (C_16_H_18_O_9_), an important bioactive substance, exhibits various functions, including antibacterial, antioxidant and antiviral activities; increasing white blood cells; protecting the liver and gallbladder; exerting anti-tumor effects; lowering blood pressure and lipids; and stimulating the central nervous system [19]. Chlorogenic acid conjugation is a safe and effective method to improve the emulsifying properties of proteins [21,22], but its effect on the emulsion gel properties of proteins is limited. The results of our preliminary experiments showed that chlorogenic acid grafting significantly improved the emulsifying activity (from 92.36 to 157.67 m^2^/g) and emulsion stability (from 85.45% to 92.37%). Brito & Silva [23] found that ultrasound can induce almond emulsion to form gels. However, the effects of combining ultrasonication and chlorogenic acid conjugation on the emulsion gel properties have been little studied.

Herein, sweet almond expeller globulin modified by ultrasonication and chlorogenic acid conjugation (SAEG-UCA) was used to prepare emulsion gels. The structural, textural, rheological, and digestive properties, and stability of the emulsion gel were studied. Moreover, the underlying mechanism by which ultrasonication and chlorogenic acid grafting improved the emulsifying and emulsion gel properties of SAEG was investigated. This study provides new and more concise strategies to enhance the performance of protein emulsion gels.

## 2. Materials and Methods

### 2.1. Materials

Sweet almonds (*Prunus armeniaca*) were harvested in Hejin, Yuncheng City, Shanxi Province, China, in June 2026. The sweet almond expeller was donated by Jinsun Almond Oil Processing Co., Hejin, China. Reagents of analytical grade, including petroleum ether, chloroacetic acid, chlorogenic acid, anhydrous ethanol, sodium hydroxide, glacial acetic acid, disodium hydrogen phosphate, anhydrous calcium chloride, methanol, pepsin (from porcine stomach mucosa, 3.0 × 10^3^ U/mg), trypsin (from pig pancreas, 1:350 U/mg), *n*-butanol, ammonium thiocyanate, sodium dodecyl sulfate, and anhydrous calcium chloride were obtained from Soliabo Chemical Co., Ltd., Tianjin, China. Curcumin (purity > 95%) was purchased from Peisi Biochemical. Co., Ltd., Changzhou, China.

### 2.2. Extraction and Ultrasonication of SAEG

Sweet almond expeller was defatted using petroleum ether II (with a boiling point of 60–90 °C) at a ratio of 1:20 (g/mL) in triplicates. The defatted sweet almond expeller was crushed and sieved through a 100-mesh sieve (TJ-224, Yushang Sieve Instrument Factory, Shangyu, China) [4]. The obtained powder (69.5 g) was mixed with 350 mL of NaCl (0.2 mol/L) and stirred at a rotation speed of 220 r/min using a YZD-V Oscillator (Dawu oscillator Co., Ltd., Xuyu, China). After 2 h, the extraction suspension was filtered and the filtrate was centrifuged at 10,150× *g* for 17 min. The supernatant was dialyzed against deionized water (dH_2_O) using a dialysis membrane with a molecular weight cutoff of 3500 Da at 4 °C for 48 h. The dH_2_O was changed every 4 h. The retentate was freeze-dried using an FID75 lyophilizer (Xinyi Dry Instrument Factory, Nantong, China), and sweet almond kernel expeller globulin (SAEG) was obtained.

Next, 10 g of SAEG was dispersed in 200 mL of NaCl (0.2 mol/L) and treated using a Ljna-J54 ultrasonic generator (Lingjiang Instrument Factory, Jingzhou, China) at 7.90 W/mL, 40 °C, and 80 kHz for 45 min [18]. The temperature was actively controlled at 40 ± 1 °C. The size parameters of the inner tank were 470 × 285 × 285 mm. The treated dispersion was collected and lyophilized using a CNMXS-009 lyophilizer (Hanxin Dryer Instrument, Jinhua, China) to obtain ultrasound treated sweet almond globulin (SAEG-U).

### 2.3. Preparation of SAEG-Chlorogenic Acid Conjugates

Equal volumes of the SAEG solution (1 g/100 mL, dissolved in 0.2 mol/L of NaCl) and chlorogenic acid solutions (5 mg/mL, pH 10.0) were mixed and stirred at a rotation speed of 140 r/min using the YZD-V oscillator [24]. After 21 h, the reaction solution was centrifuged at 4350× *g* for 15 min. The supernatant was dialyzed against dH_2_O using a dialysis membrane with a molecular weight cutoff of 3500 Da at 4 °C for 48 h. The dH_2_O was changed every 4 h [19]. The retentate was freeze-dried, and SAEG modified by ultrasonication and chlorogenic acid conjugation (SAEG-UCA) was obtained. The Folin–Ciocalteu method was employed to measure the conjugation degree [25].

### 2.4. Emulsifying and Relative Properties

#### 2.4.1. Solubility and Surface Hydrophobicity

SAEG, SAEG-U, and SAEG-UCA were separately dissolved in dH_2_O (2 g/100 mL) and then adjusted to pH values of 2.5, 4.5, 6.5, 8.5, and 10.5, respectively. The dispersions were shaken at 25 °C in an HII-7250 triangular flask (Jiangzhe Triangular Instrument factory, Jinhua, China) for 75 min [20]. The dispersions were centrifuged at 4275× *g* for 20 min, and the protein content in the supernatant was quantified using the Bradford method [26]. The percentage of the protein content in the supernatant per 2 g/100 mL was expressed as the solubility (g/100 g). Moreover, the surface hydrophobicity of SAEG, SAEG-U, and SAEG-UCA was measured using the 8-anilino-1-naphthalenesulfonic acid method following the same protocols described by Wang et al. [20].

#### 2.4.2. Interface Sorption Ability

The interface sorption ability of SAEG, SAEG-U, and SAEG-UCA at different pH levels (2–10) was measured using the centrifugation method described by Wang et al. [20]. Briefly, the emulsions prepared using SAEG, SAEG-U, and SAEG-UCA were centrifuged at 7850× *g* in triplicate, and each run took 20 min. The supernatant was pooled and used for protein content determination following the Bradford method [26]. The difference between the initial and final protein content, relative to the initial protein content, was defined as the interface sorption ability.

#### 2.4.3. Emulsifying Capacity and Emulsion Stability

The emulsifying capacity and emulsion stability of SAEG, SAEG-U, and SAEG-UCA were determined according to the turbidity method [27]. The volume ratio of the SAEG solution (1 mg/mL, dissolved in 0.2 mol/L of NaCl) to soybean oil was 3:1, and the homogenization rate and time were 11,250 r/min and 120 s, respectively. Next, 40 μL of the emulsion was drawn out and diluted 500 times using an SDS solution (1 g/100 mL). The absorbance at 500 nm of the dilution after 0 and 10 min was recorded as *A*_0_ and *A_t_*. Equations (1) and (2) were used for quantification of the emulsion stability and emulsifying ability, respectively:

(1) $$\;Emulsionstability\left(\%\right)=\;A_{t}/A_{0}\times 100\%$$

(2) $$Emulsifiability(m^{2}/g)=(2.303\times 2\times A_{0}\times f)/(C\times \varphi \times 10,000)$$

where 2 and 2.303 are constants; *f* is the dilution factor (500); *C* is the protein content; and *φ* is the oil volume fraction (0.25).

#### 2.4.4. Zeta Potential

The zeta potential of the emulsions prepared as described in Section 2.4.3 was measured using an LJ-159 Micro-Potential meter (Huiai Potential Instrument Factory, Nanjing, China) [28].

### 2.5. Preparation of Emulsion Gels

Following the method of Kamer [29], 45 mL of SAEG solution with different concentrations (final content of 4–12 g/100 g in the gels), 3 mL of sodium alginate (2 g/100 mL), and 15 mL of soybean oil were homogenized at 20,000 rpm for 2 min using an HTX-II rapid dispersion device (Hangzhou DSIRAD Co., Ltd., Hangzhou, China). Calcium chloride (5.54 mg/mL) was immediately added to form a suspension, and then homogenized at 20,000 rpm for 60 s. The homogeneous liquid was heated at 80 °C for 30 min and placed at 4 °C for 16 h, and then, the gel was formed.

### 2.6. Structural Characteristics of SAEG and the Emulsion Gels

#### 2.6.1. Morphology Analysis

The emulsion gels based on SAEG and SAEG-UCA (with content of 6–14 g/100 g) were placed upside down at room temperature for 12 h and then photographed using a Konica camera without amplification [27].

#### 2.6.2. Surface Microstructure Analysis

SAEG- and SAEG-UCA-emulsion gels (2 g) were cut into pieces with a diameter of approximately 1 cm and then dehydrated in a vacuum dryer. After 48 h, the residual oil droplets were removed by soaking them in ether. The dry emulsion gel powder was investigated using a JOLE-JMS-70E50 electron microscope (Electronics Corporation, Osaka, Japan) for apparent morphology and microstructure analyses. Briefly, the sample was pasted on conductive adhesive and then underwent gold spraying (10 nm) [28]. The magnification factor was 10,000, with an accelerating voltage of 10 kV and a scale of 1 μm.

#### 2.6.3. X-Ray Diffraction

The dry emulsion gel powder (100 mg) prepared as described in Section 2.6.2 was loaded into the sample tank of an ARL EQUINOX 100 *X*-ray diffractometer (Thermo Fisher Scientific, Waltham, MA, USA) [10]. The scanning speed, 2*θ* scanning range, and current were 10°/min, 5°–70°, and 30 mA, respectively. The lowest intensity (*I_am_*) and maximum intensity (*I*_002_) were determined and Equation (3) was used to quantify the crystallinity:

(3) $$Crystallinity\left(\%\right)=(1-I_{am}/I_{002})\times 100$$

#### 2.6.4. Fourier-Transformed Infrared (FT-IR) Spectroscopy

The SAEG-, SAEG-UCA-, and dried SAEG-emulsion gel powder (1 mg) prepared as described in Section 2.6.2 and potassium bromide (approximately 100 mg) were thoroughly mixed, ground, and then compressed into thin slices. The slice was placed on the sample loading platform of a Nicolet CCR-II FT-IR Spectrometer (Thermo Fisher Scientific, Waltham, MA, USA) and analyzed from 4000 to 400 cm^−1^. The resolution was 4 cm^−1^ [30].

#### 2.6.5. Intermolecular Interactions

Following the selective destruction method described by Han et al. [17], the fresh wet emulsion gel (1 g) was separately dissolved in four types of solution: sodium chloride solution (SA, 0.6 mol/L); 1.5 mol/L of urea solution containing 0.6 mol/L of sodium chloride (SB); 8 mol/L of urea solution containing 0.6 mol/L of sodium chloride (SC); and 8 mol/L of urea solution containing 0.6 mol/L of sodium chloride and 0.5 mol/L of β-mercaptoethanol (SD). These four kinds of suspensions were thoroughly stirred at 12,000 rpm for 50 s using the HTX-II rapid dispersion device, and centrifuged at 10,000× *g* and 4 °C for 0.5 h. The supernatant was used for protein concentration determination using the Coomassie brilliant blue assay [26]. The concentration of protein in the supernatant extracted by SA, and the protein concentration differences between SB and SA, SC and SB, and SD and SC can provide operational estimates of interaction contributions to the ionic bonds, hydrogen bonds, hydrophobic interactions, and disulfide bond contents, respectively.

### 2.7. Characteristics of the Emulsion Gels

#### 2.7.1. Water Distribution

The water distribution within the emulsion gels based on SAEG and SAEG-UCA (12 g/100 g) was analyzed using low-field nuclear magnetic resonance equipped with a Carr-Purcell–Meiboom–Gill sequence (Brook Technology Co., Ltd., Beijing, China). Nuclear magnetic resonance analysis was carried out at 32 °C. The RF delay, echo time, and number of scans were 0.5 ms, 0.5 ms, and 8, respectively [29]. The relaxation time (T_2_) and the corresponding area were calculated using the MestReNova 14 software (MestrelabResearch, Santiago de Compostela, Spain) consequently.

#### 2.7.2. Texture Properties

The texture properties of SAEG-emulsion gels, including cohesiveness, hardness, chewiness, gumminess, and resilience were determined using a TA^+^ textural analyzer (Lloyd’s Instruments, AMETEK, Inc., Wallingford, CT, USA) and a P/36 R probe [9]. The operation was carried out when the pre-, mid-, and operation rates were 2, 1, and 1 mm/s, respectively. The compression rate was 50%, and the trigger force was 3 g.

#### 2.7.3. Rheological Characteristics

The energy storage modulus (*G*′), viscosity, loss factor, and loss modulus (*G*″) of emulsion gels based on SAEG and SAEG-UCA (12 g/100 g) were investigated using an TA.XTC-20 texture analyzer (Keji Rheometer Instrument Factory, Zhenjiang, China). First, the emulsion gels were placed on a P550P parallel plate and equilibrated at 25 °C (using the T-PPD controller) for 5 min to relax loading stresses [31]. Viscosity was determined at a shear rate of 0.1–100 s^−1^. During dynamic frequency scanning, the oscillation frequency, linear range, and gap were 0.1–20 Hz, 0.1–100 rad/s, and 1 mm, respectively. The stress range was 0.1–1000 Pa. The *G*′ and *G*″ values under cooling and heating were determined, and the rates of temperature change during heating (25–100 °C) and cooling (100–25 °C) were both 10 °C/min. The loss factor (tan δ = *G*″/*G*′) was calculated. All tests were carried out in triplicate.

### 2.8. Oxidation Stability

The emulsion gels based on SAEG and SAEG-UCA (12 g/100 g) were left at 60 °C for 14 d to evaluate their oxidation stability [32]. Every two days, the SAEG-emulsion gel (0.1 g) was mixed with 1.5 mL of isooctane and 0.5 mL of an isopropanol-mixed solution. The mixture was vortexed at 1250 rpm for 15 s, and centrifuged at 3400× *g* for 120 s. Then, the upper solution (0.2 mL) was pipetted into 2.8 mL of a butanol and methanol mixture (2:1, *v*/*v*). Next, 3.9 mol/L of NH_4_SCN (15 μL) and 0.132 mol/L of FeCl_2_ (15 μL) were added and vortexed at 1250 rpm for 10 s. The absorbance of the reaction solution was recorded at 510 nm after 20 min of dark incubation. The primary lipid oxidation value was calculated with cumene hydroperoxide as the standard.

### 2.9. Preparation of the Emulsion Gels Loading with Curcumin

Based on the modified method of Gray et al. [11], curcumin was dissolved in refined corn oil (5 mg/mL), and then mixed with the SAEG solution (4.5 g/100 mL) at a volume ratio of 1:3 (*v*/*v*), along with 3 mL of sodium alginate (2 g/100 mL). The mixture was homogenized at 20,000 rpm for 120 s with the HTX-II rapid dispersion device. Immediately, calcium chloride powder was added to reach a concentration of 5.54 mg/mL. The suspension was homogenized at 20,000 rpm for 120 s, heated at 80 °C for 30 min, and then left at 4 °C for 10 h to form an emulsion gel loaded with curcumin.

### 2.10. Stability and Functionality of Curcumin in the Emulsion Gels

#### 2.10.1. Photostability of Curcumin

The emulsion gels loaded with curcumin (1 g) were exposed to UV light for 3, 6, 9, 12, 15, 18, 21, and 24 h, respectively. Subsequently, the gels were cut into pieces and immersed into 3 mL of methanol [10]. The mixture was vortexed at 3000 r/min and 25 °C for 3 min, and then centrifuged at 10,000× *g*. The absorbance of the supernatant was measured at 470 nm, and the content of curcumin was calculated according to the standard curve. The curcumin in corn oil was used as the control.

#### 2.10.2. Encapsulation Efficiency and Loading Capacity of Curcumin in the Emulsion Gels

Approximately 1 g of SAEG-emulsion gels loaded with curcumin was cut into pieces and immersed into 5 mL of anhydrous ethanol [10]. The suspension was vortexed at 3000 r/min and 30 °C to break the emulsion. After 3 min, the mixture was then centrifuged at 8000× *g*, and the supernatant was determined at 470 nm. A standard curve with curcumin was drawn, and the encapsulation efficiency, and curcumin loading capacity of curcumin were calculated using Equations (4) and (5):

(4) $$Encapsulation\;effciency\;\left(\%\right)=\;W_{2}/W_{1}\times 100\%\;$$

(5) $$Loading\;capacity\;\left(\%\right)=W_{3}/W_{2}\times 100\%$$

where *W*_1_, *W*_2_, and *W*_3_ are the curcumin weights in the sample, refined corn oil, and the curcumin-loaded emulsion gel, respectively.

#### 2.10.3. Bioaccessibility of Curcumin in the Emulsion Gels

Following the method of Gray et al. [11], the curcumin-loaded emulsion gels (approximately 4 g) were placed in a triangular flask and shaken at 3250 rpm for 12 s to simulate oral chewing. Next, the curcumin loaded emulsion gel pieces were digested by 40 mL gastric digestive juices (containing 4000 U/mL of pepsin and 2 mg/mL of sodium chloride) at pH 1.5 ± 0.1 and 37 °C for 1.5 h, and then digested by 40 mL of the simulated intestinal fluid (composed of 300 U/mL of lipase, 800 U/mL of trypsin, 9.8 mg/mL of bile sodium, and 10 mmol/L of CaCl_2_) at pH 7.0 ± 0.1 and 37 °C for 2.5 h. Here, 0.1 mol/L of NaOH was used to maintain the pH value at 7.0 ± 0.1. Subsequently, the digestive fluid was centrifuged at 4350× *g* for 20 min, and the content of curcumin in the supernatant was measured. The bioaccessibility of curcumin was calculated using Equation (6):

(6) $$Bioaccessiblity\;of\;curcumin\;\left(\%\right)=W_{2}/W_{1}\times 100\%\;\;$$

where *W*_2_ and *W*_1_ are the curcumin in the emulsion gel and the supernatant, respectively.

### 2.11. Statistical Analysis

The repeat time for every test was three or more. Statistical analysis was conducted using Version 16 IBM SPSS Statistics software (Chicago, IL, USA); significance and post hoc multiple comparisons were performed with one-way analysis of variance (ANOVA), factorial ANOVA, and Tukey’s honestly significant difference test, respectively, and the minimum number of independent replicates was 3 times. The significant level was set at *p* < 0.05.

## 3. Results and Discussion

### 3.1. The Degree of Substitution and FT-IR Spectroscopy of SAEG-UCA

**Figure 1:**
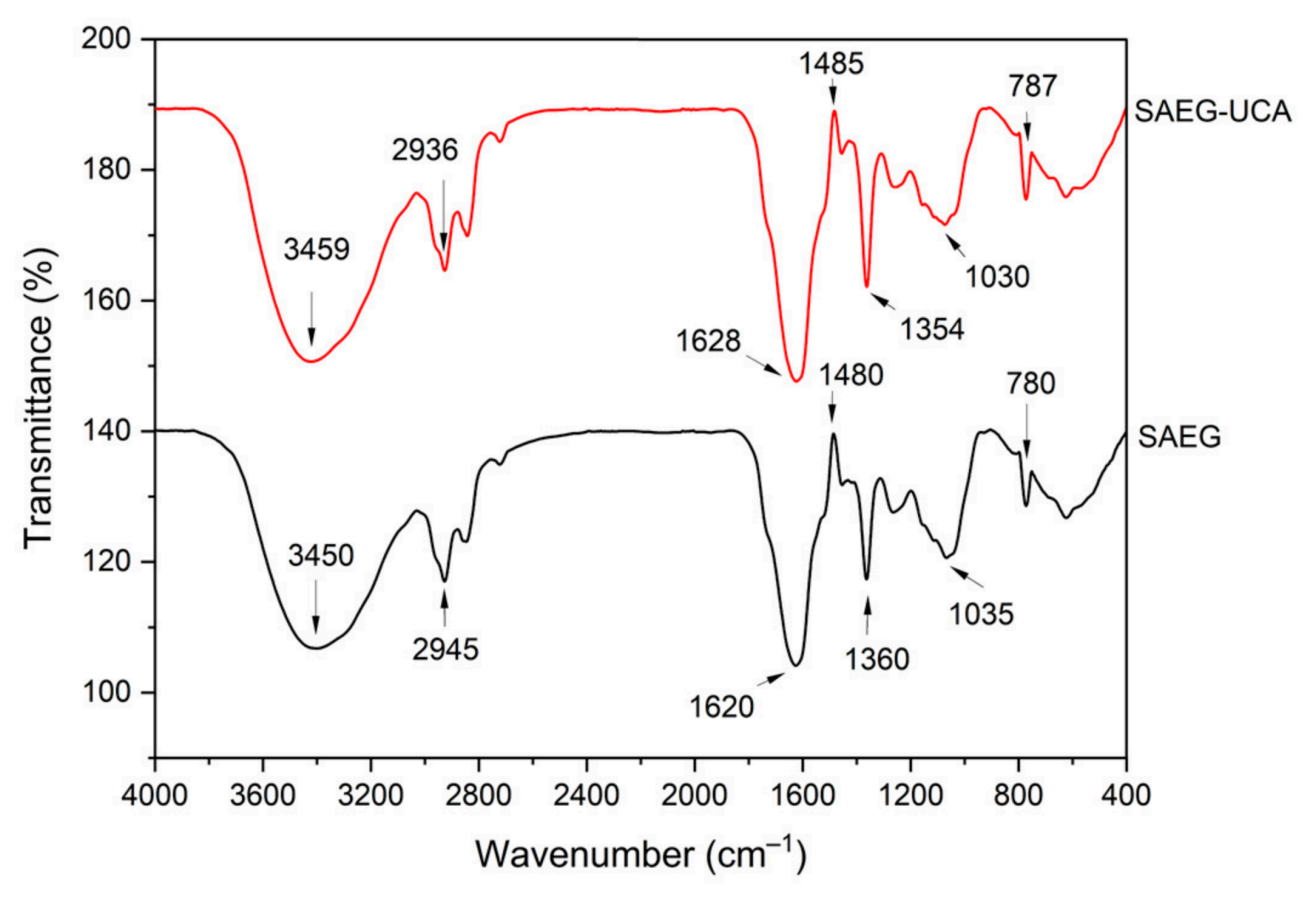
The Fourier-transform infrared spectroscopy of SAEG and SAEG-UCA.

The degree of substitution of SAEG-UCA was 7.84% ± 0.47%, indicating that chlorogenic acid groups have been bound to SAEG. As shown in Figure 1, compared to the FT-IR spectrum of SAEG, the peak at 3450 cm^−1^ (indicative of the stretching vibration peak of N–H or –OH bond) shifted to 3459 cm^−1^ in the spectrum of SAEG-UCA. The peak at 1620 cm^−1^ (corresponding to the C=O bond’s asymmetric stretching) shifted to 1628 cm^−1^ after ultrasonication and chlorogenic acid conjugation, suggesting that these modifications affected the amide bands I and II of MCP [33]. Moreover, a visible blue shift from 780 to 787 cm^−1^ (attributed to C–O–C vibration) was observed in the spectrum of SAEG-UCA [31], confirming the degradation caused by ultrasonication [18]. Furthermore, compared to the FT-IR spectra of SAEG, obvious shifts appeared at 1480 and 1360 cm^−1^, corresponding to the C–O stretching and the aromatic ring vibrations, respectively, and indicating that chlorogenic acid groups have been successfully grafted onto SAEG [24].

### 3.2. The Solubility and Surface Hydrophobicity of SAEGs

**Figure 2:**
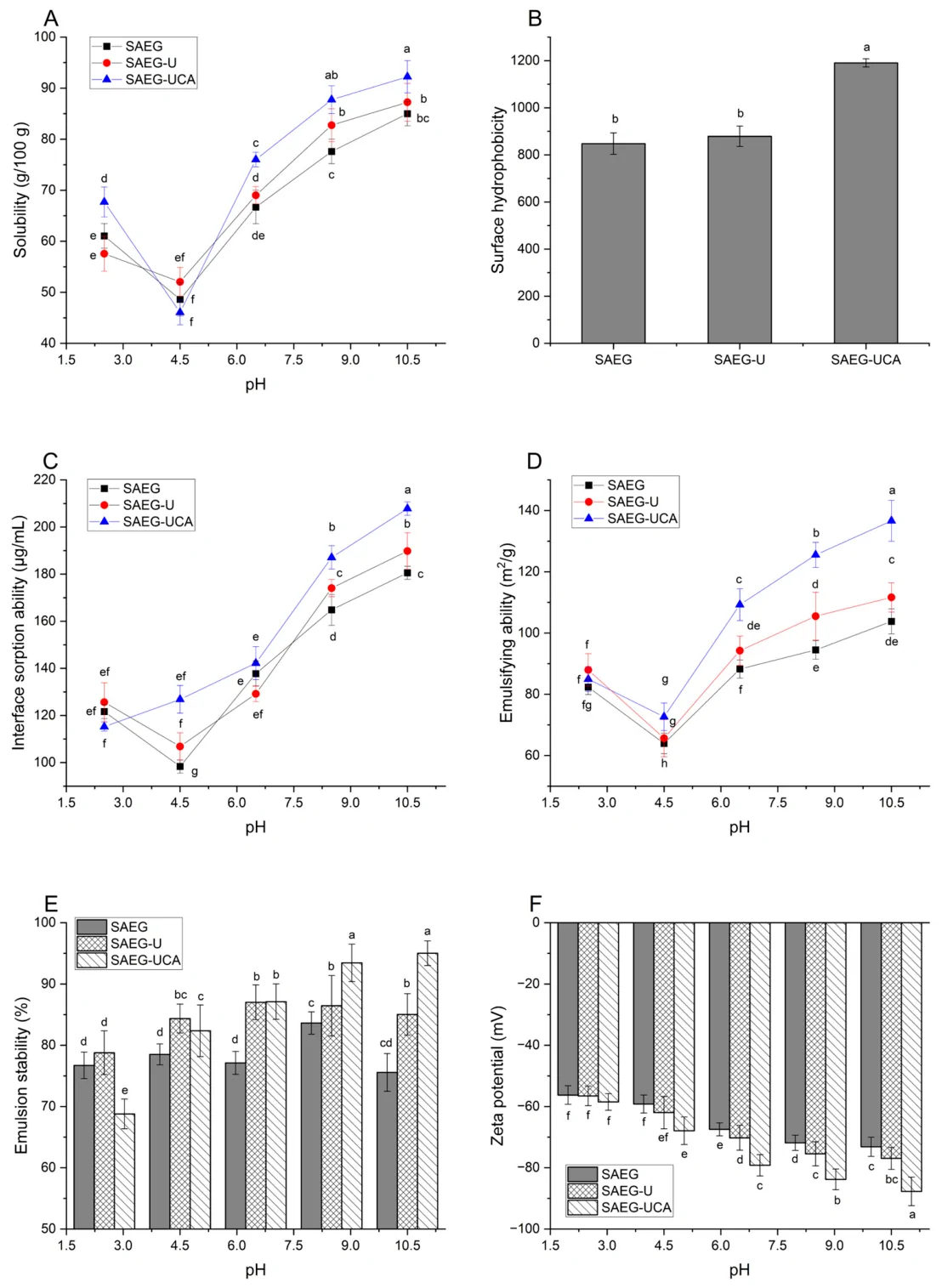
The solubility (**A**), surface hydrophobicity (**B**), interface sorption capacity (**C**), emulsifying capacity (**D**), and emulsion stability (**E**) of SAEG, SAEG-U, and SAEG-UCA; and the zeta potential (**F**) of the emulsions based on SAEG and SAEG-UCA. SAEG, sweet almond expeller globulin; SAEG-U, sweet almond expeller globulin modified by ultrasonication; SAEG-UCA, sweet almond expeller globulin modified by ultrasonic and chlorogenic acid conjugation. Different lowercase letters (a–h) near the bars or data points indicate statistically significant differences (*p* < 0.05).

As shown in Figure 2A, SAEG-U showed a higher solubility than SAEG at pH 4.5 and pH 7.5, but its solubility was lower than that of SAEG-UCA at pH values of 2.5, 6.5, and 10.5 (*p* < 0.05), indicating that ultrasonication increased the hydrophilicity of SAEG and that combining ultrasonication and chlorogenic acid conjugation was more effective in improving the solubility of SAEG than ultrasonication alone. Ultrasonic treatment caused a breakdown of the amino bonds within polypeptides and exposed more hydrophilic groups [33]; in addition, the –OH in the introduced chlorogenic groups was hydrophilic [19], enhancing the solubility of SAEG. Moreover, SAEG, SAEG-U, and SAEG-UCA exhibited increasing solubility when pH values increased from 4.5 to 10.5, with the lowest solubility observed at pH 4.5, which was close to the isoelectric point of almond globulin [6]. At the isoelectric point, the net charge of SAEG was nearly zero and the repulsive forces between protein molecules were low [20], resulting in a poor solubility. As the pH increased, the charged groups gradually dissociated, and the net charge increased. The water-binding capacity of SAEG was enhanced, which may have thickened the hydration membrane surrounding SAEG molecules, resulting in a superior solubility [23].

Moreover, ultrasonication and chlorogenic acid conjugation increased the surface hydrophobicity of SAEG (*p* < 0.05) (Figure 2B). Ultrasonication exposed more hydrophilic groups from SAEG; the introduced chlorogenic acid increased its steric hindrance and expanded the structure of SAEG; the benzene ring group and quinic acid residue in chlorogenic acid were hydrophobic [22], resulting in a higher surface hydrophobicity.

### 3.3. The Interface Sorption Ability of SAEGs

As shown in Figure 2C, the interface sorption ability of SAEG-UCA was higher than that of SAEG and SAEG-U at pH 4.5–10.5, and SAEG-U showed higher interface sorption ability than SAEG at pH 4.5, 8.5 and 10.5 (*p* < 0.05), demonstrating that ultrasonication increased the interface sorption ability of SAEG. Moreover, ultrasonication combined with chlorogenic acid conjugation was more effective in increasing the interface sorption ability compared with ultrasonication alone. Ultrasonication and chlorogenic acid conjugation exposed more hydrophilic and hydrophobic groups from SAEG (Figure 2A,B) and increased the steric hindrance, which can render its structure more flexible, thereby enhancing its adsorbing ability at the water–oil interface [32]. Furthermore, the benzene ring group and quinic acid residue in chlorogenic acid were hydrophobic [22], increasing the affinity of SAEG to oils and resulting in a higher interface sorption ability.

### 3.4. Emulsifying Properties of SAEGs

SAEG exhibited lower emulsion stability and emulsifying capacity than SAEG-U and SAEG-UCA (*p* < 0.05) (Figure 2D,E); this demonstrates that reflecting ultrasonication and ultrasonication assisted with chlorogenic acid conjugation both improved the emulsifying properties of SAEG (*p* < 0.05). Ultrasonication and chlorogenic acid conjugation increased the solubility and surface hydrophobicity of SAEG (Figure 2A,B) and further enhanced its binding affinity to water and oils at the water–oil interface, thereby improving its emulsifying capacity. Moreover, it was reported that an increase in interface sorption capacity promoted the formation of a stable film surrounding the droplets [22], thereby making the emulsion more stable. In addition, the emulsions based on SAEG-U and SAEG-UCA exhibited a superior zeta potential than that of SAEG-emulsion (Figure 2F), indicating that SAEG-U and SAEG-UCA can form more stable film surrounding the droplets. The number of ions in the double layer was greater, and the films were thicker, thereby enhancing the repulsive forces between the droplets and inhibiting droplets to aggregation [5]; taken together, this phenomenon results in a better emulsion stability. Furthermore, the emulsifying capacity and emulsion stability of SAEG, SAEG-U, and SAEG-UCA both increased as the pH was raised from 4.5 to 10.5, mainly ascribed to the increased solubility and interface sorption ability (Figure 2A,C). Wang et al. [20] demonstrated that alkali pH shifts expanded the structure of plant proteins, exposed more functional groups, and promoted dissociation, resulting in an improvement in emulsifying capacity and emulsion stability. Additionally, SAEG-UCA exhibited higher emulsifying ability and emulsion stability than SAEG-U within the pH range of 4.5 to 10.5 (*p* < 0.05), showing that compared with ultrasonication, ultrasonication combined with chlorogenic acid conjugation more effectively improved the emulsifying properties.

### 3.5. Zeta Potential and Particle Size of the SAEG Emulsions

As depicted in Figure 2F, within a pH range of 4.5–10.5, the SAEG-UCA-emulsion showed higher zeta potential than the SAEG- and SAEG-U-emulsions (*p* < 0.05); moreover, the zeta potential of the SAEG-U-emulsion was higher than that of the SAEG-emulsion at pH 6.5 and 8.5 (*p* < 0.05), indicating that ultrasonication assisted with chlorogenic acid conjugation was better at increasing the zeta potential of SAEG than simple ultrasonication. Ultrasonication and chlorogenic acid conjugation enhanced the interfacial sorption ability and emulsifying capacity of SAEG (Figure 2C,D), which can increase its ability to form emulsion films surrounding the oil droplets, increase the repulsive forces between the droplets and hinder their aggregation [10,20], resulting in higher zeta potential values. Moreover, the higher solubility of SAEG-UCA and SAEG-U (Figure 2A) indicated that more proteins can take part in stabilizing the emulsion, and the number of ions in the double layer was greater, thereby enhancing the zeta potential and reducing the droplet size.

The results in Figure 2C–F showed that the combined effects of ultrasonication and chlorogenic acid conjugation on the emulsifying properties of SAEG were greater than those of ultrasonication alone; thus, SAEG-UCA was used to prepare emulsion gels and the effects of ultrasonication combined with chlorogenic acid conjugation on the emulsion gel properties of SAEG were investigated.

### 3.6. Structural Characteristics of Emulsion Gels

#### 3.6.1. Morphology and Electron Microscopy

**Figure 3:**
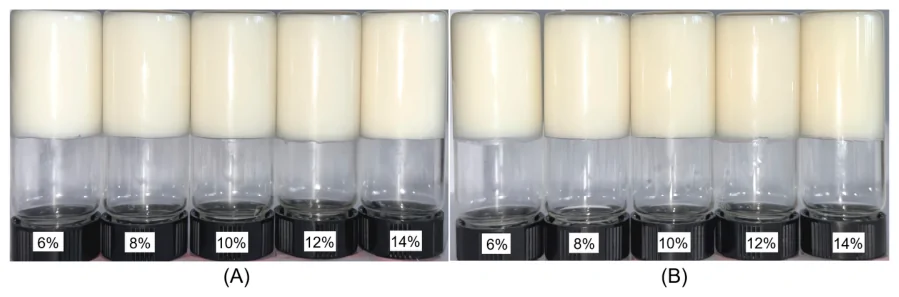
The morphologies of the emulsion gels based on SAEG (**A**) and SAEG-UCA (**B**) after storage for 12 h. The picture is without magnification.

**Figure 4:**
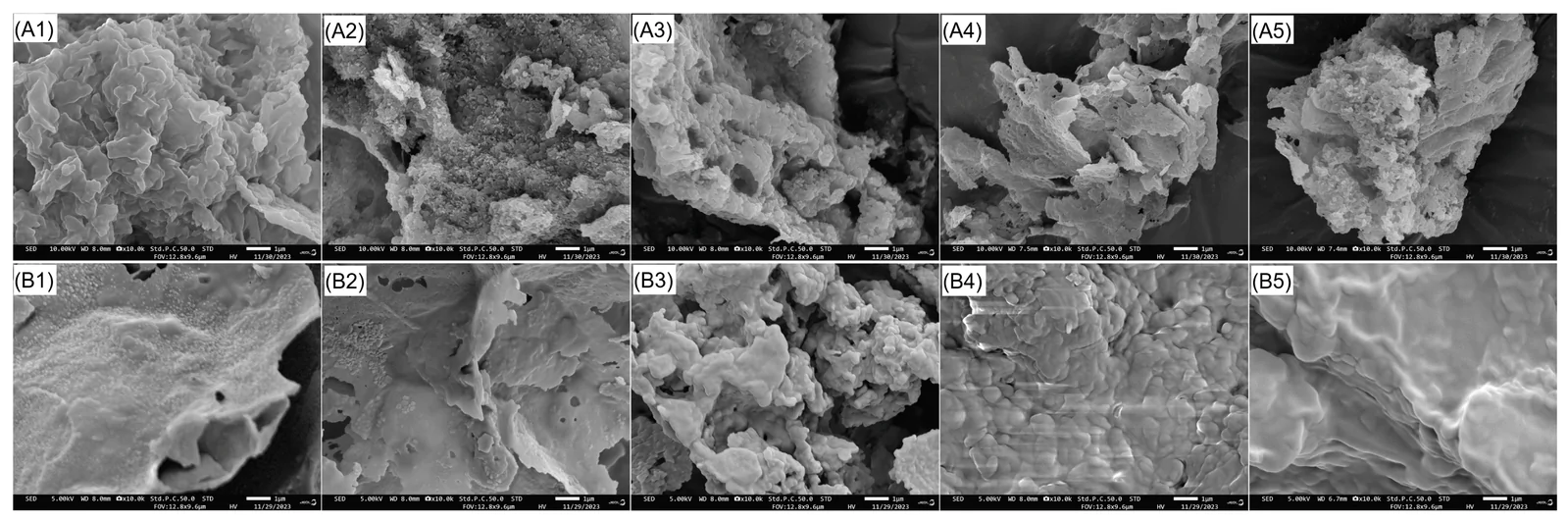
The scanning electron micrographs of the emulsion gels based on SAEG (**A1**–**A5**) and SAEG-UCA (**B1**–**B5**) at addition amounts of 6–14 g/100 g. The magnification for scanning electron micrographs was 10,000× with a scale bar of 1 μm.

As shown in Figure 3A,B, compact emulsion gels were formed based on SAEG and SAEG-UCA at concentrations of 6–14 g/100 g. After 12 h of inversion, the gels remained stable without any collapse or layering phenomena, and this is mainly ascribed to the excellent emulsifying capacity and emulsion stability of SAEG and SAEG-UCA (Figure 2D,E). During emulsion preparation, SAEG and SAEG-UCA rapidly adsorbed at the water–oil interface and formed stable films surrounding the droplets. The emulsion system then formed a stable gel network structure after heating and cooling [34]. Moreover, as the concentration of SAEG and SAEG-UCA increased, the gel structure of SAEG- and SAEG-UCA-emulsion gels became denser and more compact, as shown in Figure 4(A1–B5).

As depicted in Figure 4(A1–B5), the microstructure of SAEG-emulsion gels was dense and compact, with fragments and particles piling up on the surface. Moreover, its structure became more uneven and porous as the concentration increased from 8 to 14 g/100 g. As the dose increased, more SAEG could participate in the formation of the gel network structure, increasing the thickness of the film surrounding the oil droplets, and enhancing the interactions between proteins and water [35], which were conducive to the formation of a cross-linked network structure [10]. This network structure retained more water and resulted in a more porous surface microstructure after water evaporation [5].

By contrast, the microstructure of SAEG-UCA-emulsion gels was denser and smoother than SAEG-emulsion gels at addition levels of 6–14 g/100 g (Figure 4(B1–B5)), suggesting that ultrasonication and chlorogenic acid conjugation significantly enhanced the ability of SAEG to form stable emulsion gels. After ultrasonication and chlorogenic acid conjugation, the hydrophobicity, water–oil interface sorption capacity and emulsion stability of SAEG increased (Figure 2B,C,E), while the intermolecular forces within the gel were enhanced (Table 1), promoting the formation of a more stable and compact emulsion gel. Furthermore, the –OH and the benzene ring groups and quinic acid residues in chlorogenic acids can enhance the affinity of SAEG for water and oils, respectively, resulting in a more compact gel network structure [21].

#### 3.6.2. FT-IR Spectroscopy

**Figure 5:**
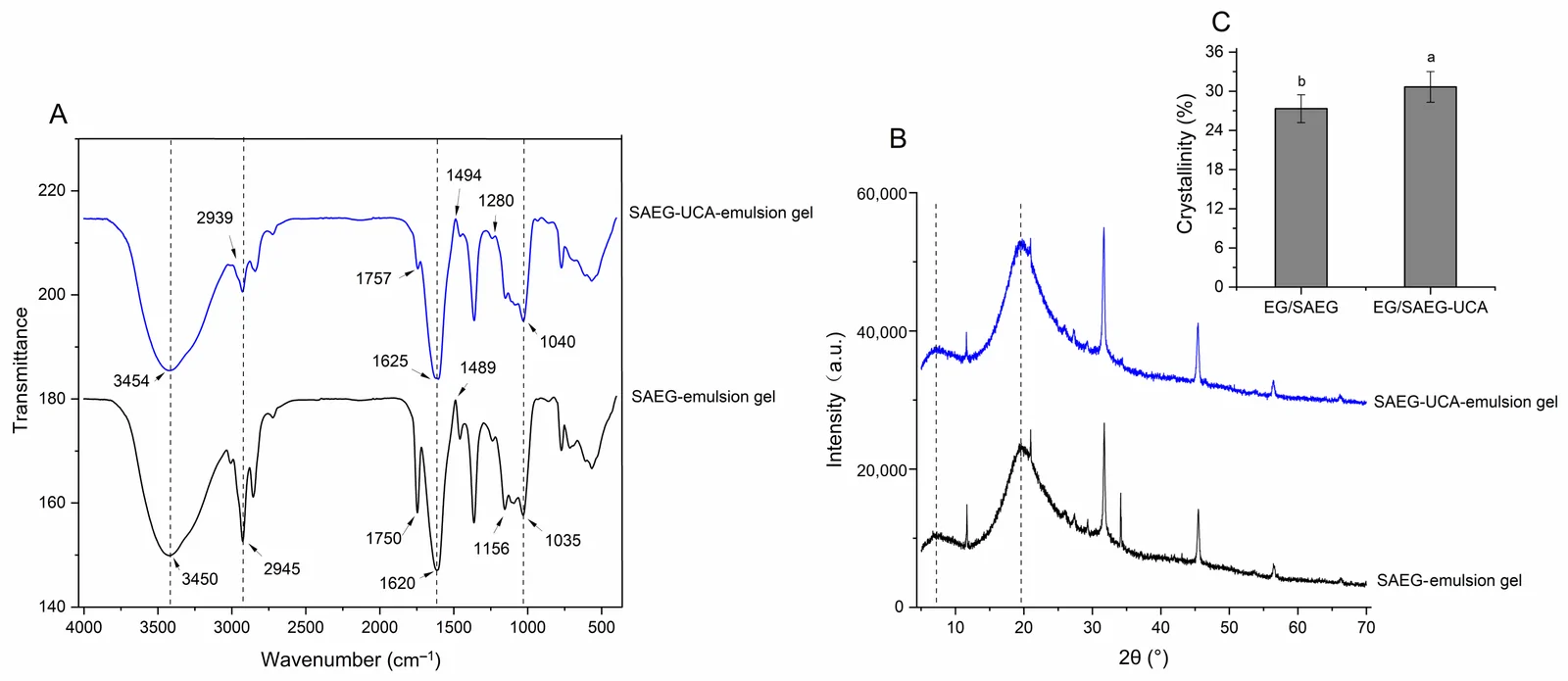
The structural characteristics of the emulsion gels based on SAEG and SAEG-UCA at concentration of 12 g/100 g. (**A**) Fourier-transform infrared spectroscopy; (**B**) *X*-ray diffraction patterns and (**C**) crystallinity. EG/SAGE, emulsion gel based on SAGE; EG/SAGE-UCA, emulsion gel based on SAEG-UCA. Different small letters (a, b) above on bars mean significant differences (*p* < 0.05).

In the FT-IR spectra of SAEG- and SAEG-UCA-emulsion gels (Figure 5A), the absorption peaks at approximately 3450, 1750, and 1035 cm^−1^ were a result of shaking –OH (or N–H), C–N, and carboxyl groups, respectively [21,36], indicating that the amide bond, hydrogen bonds, and carboxyl and amino groups in the polypeptide chains were altered during ultrasonication and heating processes, and the structure became extended; subsequently, new hydrogen bonds between protein chains and water may have formed, and a network gel structure was formed during the subsequent cooling process [10]. Moreover, ultrasonication and chlorogenic acid conjugation increased the hydrophilic regions of the gel, promoting the formation of an amphiphilicity gel. Furthermore, in the FT-IR spectra of SAEG- and SAEG-UCA-emulsion gels, the peaks at 2945, 1620, and 1489 cm^−1^ shifted to 2939, 1625, and 1494 cm^−1^, and this is ascribed to the stretching vibrations of C–H and C=C in aromatic hydrocarbons, respectively, corresponding to the introduction of chlorogenic acid [5]. Similar results were obtained by Tang et al. [19].

#### 3.6.3. XRD Analysis of Emulsion Gels

The results in Figure 5B demonstrated that SAEG- and SAEG-UCA-emulsion gels had a semi-crystalline structure and the amorphous structure was predominant, as they showed two distinct crystallization peaks at 2*θ* = 9° and 20°, respectively [24]. The SAEG-UCA-emulsion gel showed a superior crystallinity compared to SAEG-emulsion gels (*p* < 0.05) (Figure 5C), perhaps due to the introduced chlorogenic acid groups. Hydrogen bond is one key force that forms and maintains the crystal structure of emulsion gels [14]. The –OH groups in chlorogenic acids increased the affinity of SAEG-UCA for water or sodium alginate, which facilitated the formation of more intermolecular hydrogen bonds, resulting in a higher crystallinity. Coincidentally, gallic acid and ferulic acid conjugation increased the crystallinity of emulsion gels based on quinoa protein and millet prolamine, respectively [20,22].

#### 3.6.4. Intermolecular Forces Distribution

The data in Table 1 provide operational estimates of interaction contributions to the ionic bond, hydrogen bond, hydrophobic interactions, and disulfide bond within SAEG-emulsion gels, respectively, rather than direct quantitative measurements of individual molecular bonds. The disulfide bond and hydrophobic interactions were the main intermolecular interactions within the emulsions based on SAEG and SAEG-UCA. Almond globulin is a hexamer, consisting of six subunits that are covalently linked by disulfide bonds. The stability of the entire hexamer structure depends on disulfide bonds and non-covalent interactions [35]; therefore, disulfide bonds and hydrophobic interactions were the main forces maintaining the emulsion gels. Furthermore, the disulfide bonds, hydrophobic forces, and hydrogen bonds increased as the concentration of SAEGs increased (*p* < 0.05). Compared with SAEG-emulsion gels, SAEG-UCA-emulsion gels exhibited higher content of disulfide bonds, hydrophobic interactions, and hydrogen bonds at concentrations of 8–14 g/100 g. The linkages between polypeptides of SAEG may be changed after ultrasonication, chlorogenic acid conjugation, and heat processing, and some hydrophobic or hydrophilic groups, such as sulfhydryl, carboxyl and hydroxyl groups were exposed [23]. These exposed groups can form disulfide bonds, hydrogen bonds, and hydrophobic interactions among polypeptides during gel formation [8]. Moreover, the introduced chlorogenic acid improved the hydrophobicity of SAEG (Figure 2C), thereby increasing the hydrophobic interactions within SAEG-UCA-emulsion gels. The stronger intermolecular forces of SAEG-UCA-emulsion gels were one of the main reasons for their more stable and denser gel structure (Figure 3 and Figure 4), higher crystallinity (Figure 5C), and superior textural properties (Table 2).

### 3.7. Water Distribution Within the Gels

**Figure 6:**
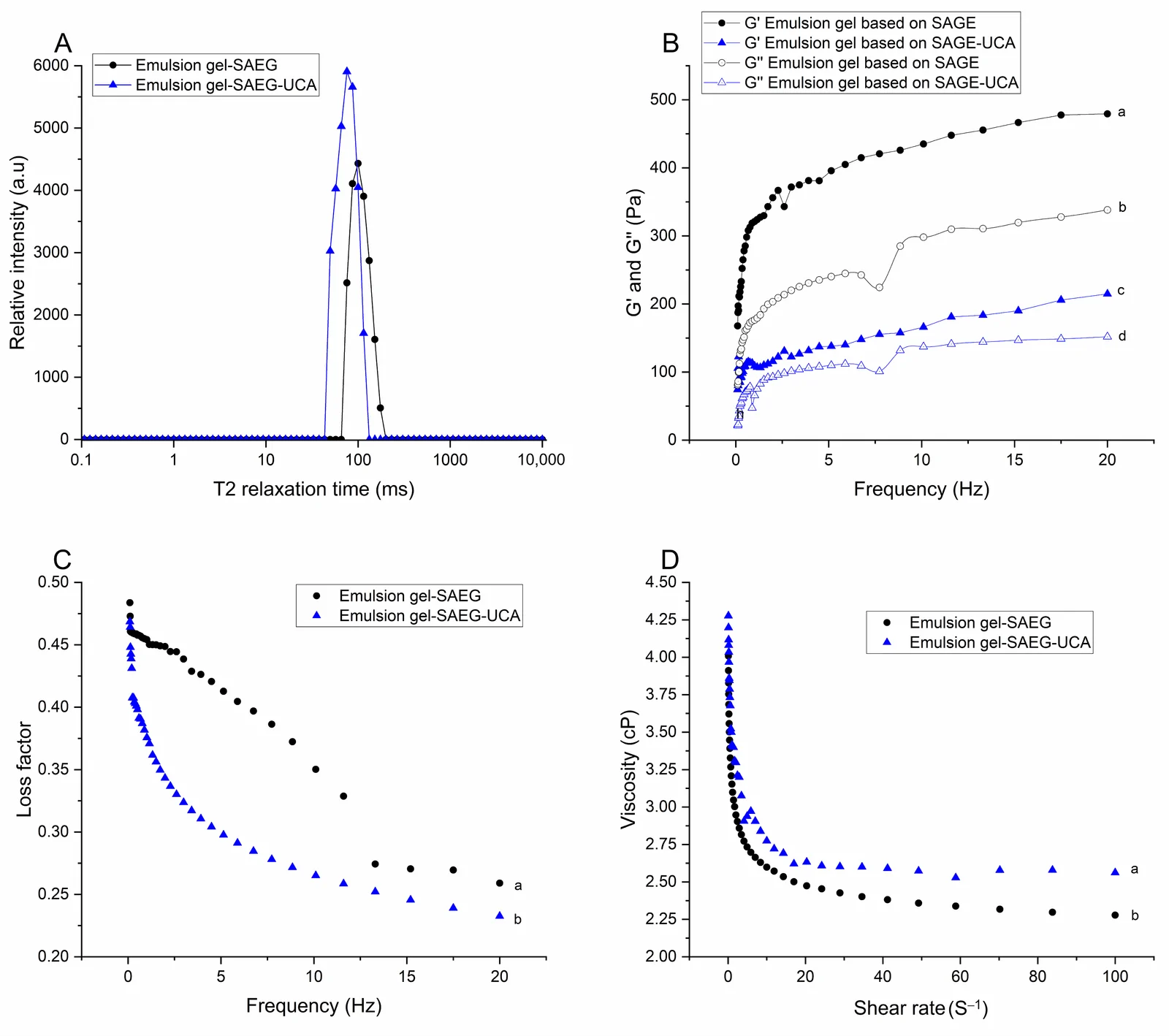
The water distribution (**A**) and rheological properties of the emulsion gels based on SAEG and SAEG-UCA with concentration of 12 g/100 g. (**B**) The energy storage modulus (*G*′) and loss modulus (*G*″), and (**C**) loss factor at various frequency; and (**D**) viscosity at different shear rate. Different lowercase letters (a–d) on the bars indicate statistically significant differences (*p* < 0.05).

As shown in Figure 6A, the SAEG-emulsion gel contained bound water (represented by the T_22_ (1–100 ms) peak area, and free water that was defined as the T_23_ (>100 ms) peak area, [10] and free water was dominant. Compared to SAEG-emulsion gels, SAEG-UCA-emulsion gels exhibited a larger T_22_ peak area but a lower T_23_ peak area, indicating that ultrasonication and chlorogenic acid conjugation increased the binding affinity of SAEG-emulsion gel to water. The reason for this phenomenon was that SAEG-UCA formed a compact and stable network gel structure (Figure 4), which can retain more water. Moreover, ultrasonication and chlorogenic acid conjugation improved the solubility and interface sorption ability of SAEG (Figure 2A,C), thereby increasing the affinity of the emulsion gel to solidify water [32]. The introduced phenolic hydroxyl groups in chlorogenic acid increased the hydrogen bonds within SAEG-UCA-emulsion gels (Table 1), which was conducive to binding and retaining water in the gel structure [8]. The transition of free water to bound water indicates an improvement in textural properties and the stability of emulsion gels.

### 3.8. Texture Properties of the Gels

The data in Table 2 revealed that ultrasonication and chlorogenic acid conjugation improved the textural properties of SAEG-emulsion gels, especially their hardness, gumminess, and chewiness. SAEG- and SAEG-UCA-emulsion gels exhibited superior hardness, springiness, gumminess, and chewiness than the SAEG-emulsion gel (*p* < 0.05). After ultrasonication and chlorogenic acid conjugation, the interface sorption ability and emulsion stability of SAEG increased (Figure 2C,E), which can promote the formation of a denser and more stable gel network structure (Figure 4(B1–B5)), thereby increasing the hardness and cohesiveness of SAEG-emulsion gels. Furthermore, ultrasonication and chlorogenic acid conjugation enhanced the intermolecular forces (hydrophobic effect and disulfide bonds) within the gel (Table 1), further improving its cohesiveness. Since the gumminess of gels is the product of hardness and cohesiveness, and chewiness is the product of gumminess and springiness [9], the increase in hardness and cohesiveness of the SAEG-emulsion gel resulted in superior gumminess and chewiness. In addition, the denser network gel structure (Figure 3) and higher bond water content (Figure 5A) are attributed to the superior springiness of SAEG-UCA-emulsion gel [12]. Alternatively, the resilience among SAEG- and SAEG-UCA-emulsion gels was not significantly different (*p* > 0.05). Apart from that, SAEG-UCA-emulsion gels exhibited higher hardness (78.63 g), cohesiveness (0.72), and chewiness (32.35 g) than those of the egg white protein emulsion gel [10], indicating their potential as food texture improvers.

The highest hardness, chewiness, and gumminess values of the SAEG- and SAEG-UCA-emulsion gels were observed when the concentration was 14 g/100 g, and their lowest values were found at 6 g/100 g, showing that their textural properties were positively dose-dependent. When the dose of SAEG and SAEG-UCA increased, the microstructure of the emulsion gels became denser (Figure 4(A1–B5)), and their abound water content, hydrophobic interactions, and disulfide bonds were enhanced (Figure 6A and Table 1), resulting in a higher hardness, cohesiveness, and springiness [31]. Furthermore, an increase in hardness and cohesiveness resulted in an increase in the chewiness and gumminess of SAEG- and SAEG-UCA-emulsion gels.

### 3.9. Rheological Properties of SAGE-Emulsion Gels

A lower loss factor (*G*″/*G*′) indicates that the emulsion gel has greater stability against reversible and irreversible deformation [23]. As shown in Figure 5B, the energy storage modulus (*G*′) and loss modulus (*G*″) of SAEG- and SAEG-UCA-emulsion gels increased as the frequency increased from 0 to 20 Hz. The *G*″ was lower than *G*′ and the loss factor value was always less than 0.5 (Figure 6C), suggesting that these emulsion gels had semisolid-like network structures [31]. Furthermore, SAEG-UCA-emulsion gels exhibited a lower loss factor than the SAEG-emulsion gel, revealing that ultrasonication and chlorogenic acid conjugation improved the rheological stability of the SAEG-emulsion gel [10]. Ultrasonication combined with chlorogenic acid conjugation rendered the network structure of the SAEG-emulsion gel (Figure 3), enhanced its intermolecular forces (Table 1), and increased its hardness, cohesiveness, and springiness (Table 2), thereby improving its rheological stability.

Moreover, the viscosity of the gels lowered as shear rates increased (Figure 6D), and the SAEG-UCA-emulsion gel exhibited higher viscosity than the SAEG-emulsion gel within a shear rate of 1–100 S^−1^, mainly ascribed to the higher crystallinity (Figure 5C) and bound water content of the SAEG-UCA-emulsion gel (Figure 6A), its stronger disulfide bonds and hydrophobic effects (Table 1), and its superior cohesiveness and gumminess (Table 2). An increase in intermolecular forces improved the cohesiveness and gumminess of the emulsion gels, thereby enhancing their structural stability and elasticity against external forces [24]. Coincidentally, the rheological properties of quinoa protein- and egg white protein-emulsion gels were improved using chlorogenic acid and tannic acid conjugation, respectively [10,19].

### 3.10. Oxidation Stability of SAEG-Emulsion Gels

**Figure 7:**
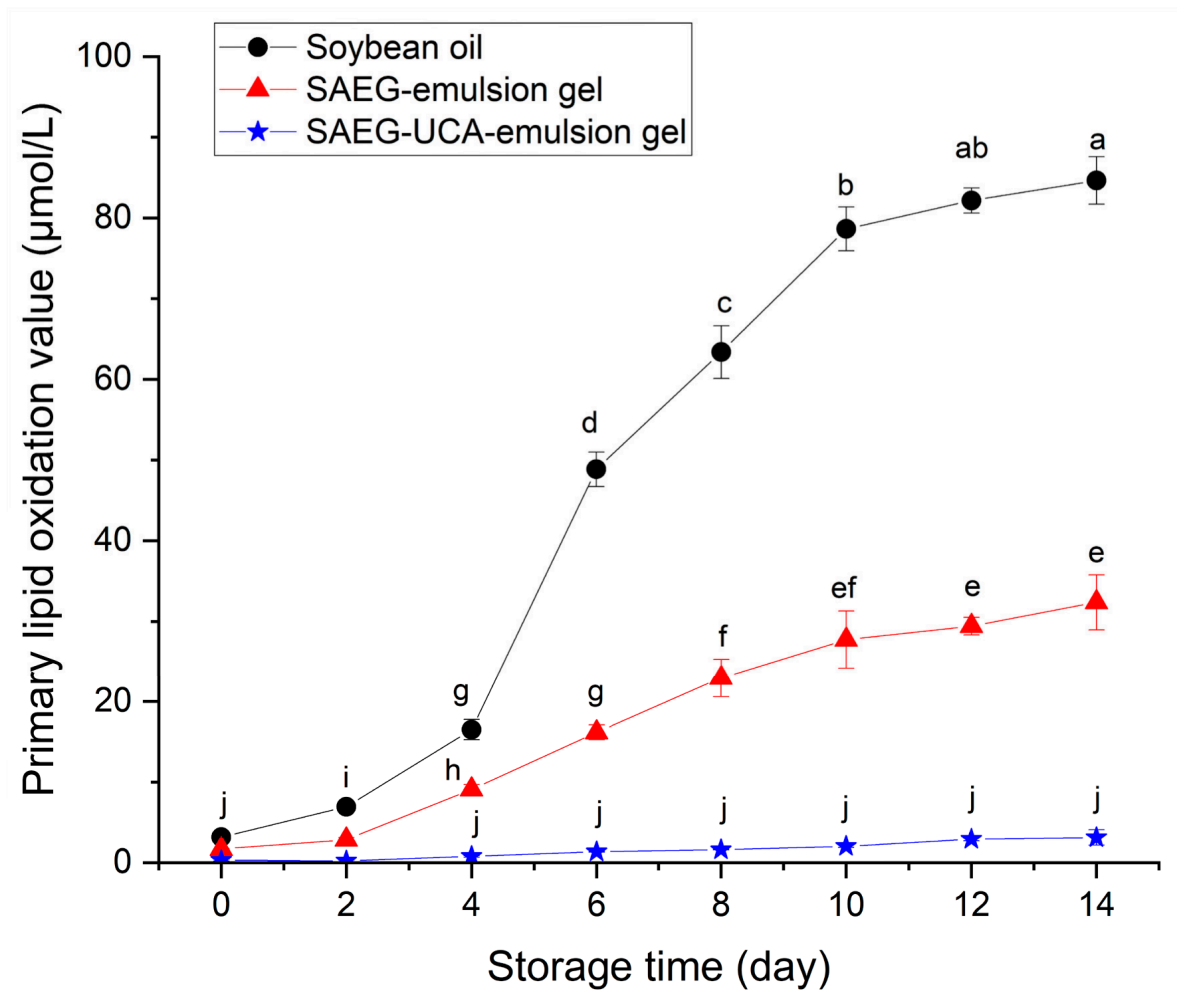
The primary lipid oxidation values of the emulsion gels based on SAEG and SAEG-UCA during storage. Different lowercase letters (a–j) on the bars indicate statistically significant differences (*p* < 0.05).

The results in Figure 7 showed that ultrasonication and chlorogenic acid conjugation remarkably enhanced the oxidative stability of SAEG-emulsion gels, as the primary lipid oxidation value of the SAEG-UCA-emulsion gel was lower than that of the SAEG-emulsion gel from the second day (*p* < 0.05). It has been proven that the phenolic hydroxyl groups in chlorogenic acid have excellent antioxidant activities [19]. Therefore, chlorogenic acid conjugation enhanced the free radical scavenging activities and reduced the power of SAEG, resulting in improved oxidative stability. Prior studies found that phenolic acid conjugation improved the oxidative stability of other emulsion gels [10,21]. Moreover, SAEG- and SAEG-UCA-emulsion gels exhibited lower primary lipid oxidation values than corn oil (*p* < 0.05), indicating that these emulsion gels effectively hindered the oxidation of lipids. First, the physical protection effect of the gels’ compact and stable network structure can scatter the light and reduce the contact rate between oxygen and the lipids. Second, some amino acid residues, such as Arg, Phe, and Tyr in almond globulin can quench free radicals and inhibit the oxidation of lipids [37].

### 3.11. Photostability of Curcumin in the Emulsion Gels

**Figure 8:**
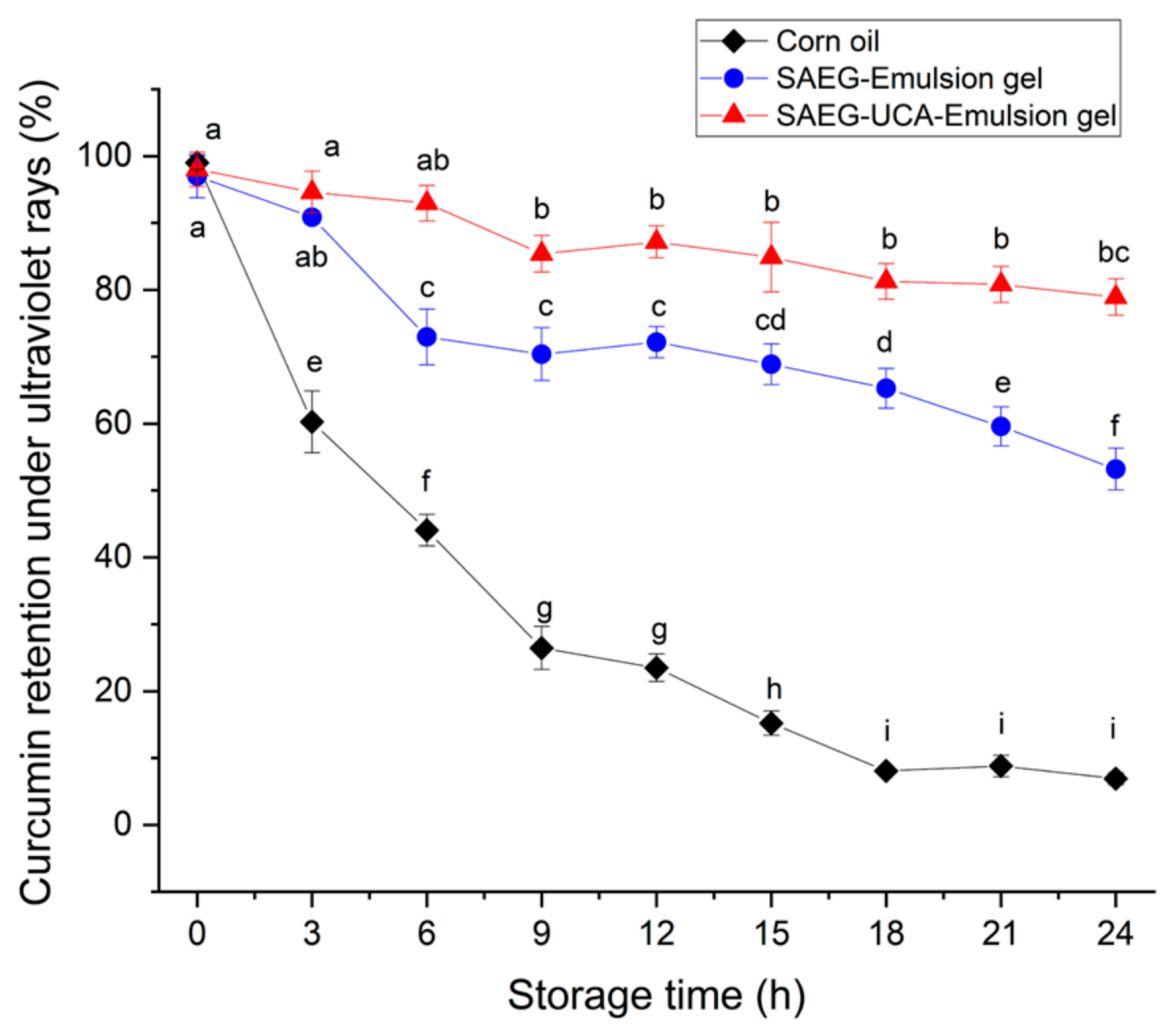
The photostability curcumin in corn oil and the emulsion gels based on SAEG and SAEG-UCA. Different lowercase letters (a–i) on the data points denote statistically significant differences (*p* < 0.05).

Curcumin is unstable under ultraviolet rays, which is one reason for its poor bioaccessibility [11]. Figure 8 shows that curcumin’s photostability in SAEG- and SAEG-UCA-emulsion gels was better than in corn oil (*p* < 0.05). The curcumin in corn oil was directly exposed to ultraviolet rays, which easily resulted in decomposition and a lower retention [31]. Alternatively, there were three main reasons for the better photostability of curcumin in SAEG-emulsion gels: (I) The oil droplets containing curcumin were surrounded by dense SAEG and SAEG-UCA, which can effectively shade the ultraviolet rays [21], thereby reducing the decomposition of curcumin; (II) SAEG- and SAEG-UCA-emulsion gels exhibited considerable antioxidant activity (Figure 7), effectively inhibiting the oxidation of curcumin caused by ultraviolet rays; and (III) the emulsion gels based on SAEG and SAEG-UCA exhibited high hardness, cohesiveness, and WHC (Table 2 and Figure 6), providing a physical barrier that can scatter ultraviolet rays. In addition, water in emulsion gels can scatter lights [13]. Begum et al. [38] found that the emulsion gels based on alginate improved the encapsulation capacity of curcumin. Moreover curcumin’s photostability in SAEG-UCA-emulsion gels was higher than in SAEG-UCA-emulsion gels from days 10 to 16 (*p* < 0.05), which was attributed to the denser compact structure (Figure 3), stronger intermolecular forces (Table 1), higher bound water content (Figure 6), and superior antioxidant activity of the SAEG-UCA-emulsion gel (Figure 7).

### 3.12. Encapsulation, Loading Capacities, and Bioaccessibility of Curcumin

**Figure 9:**
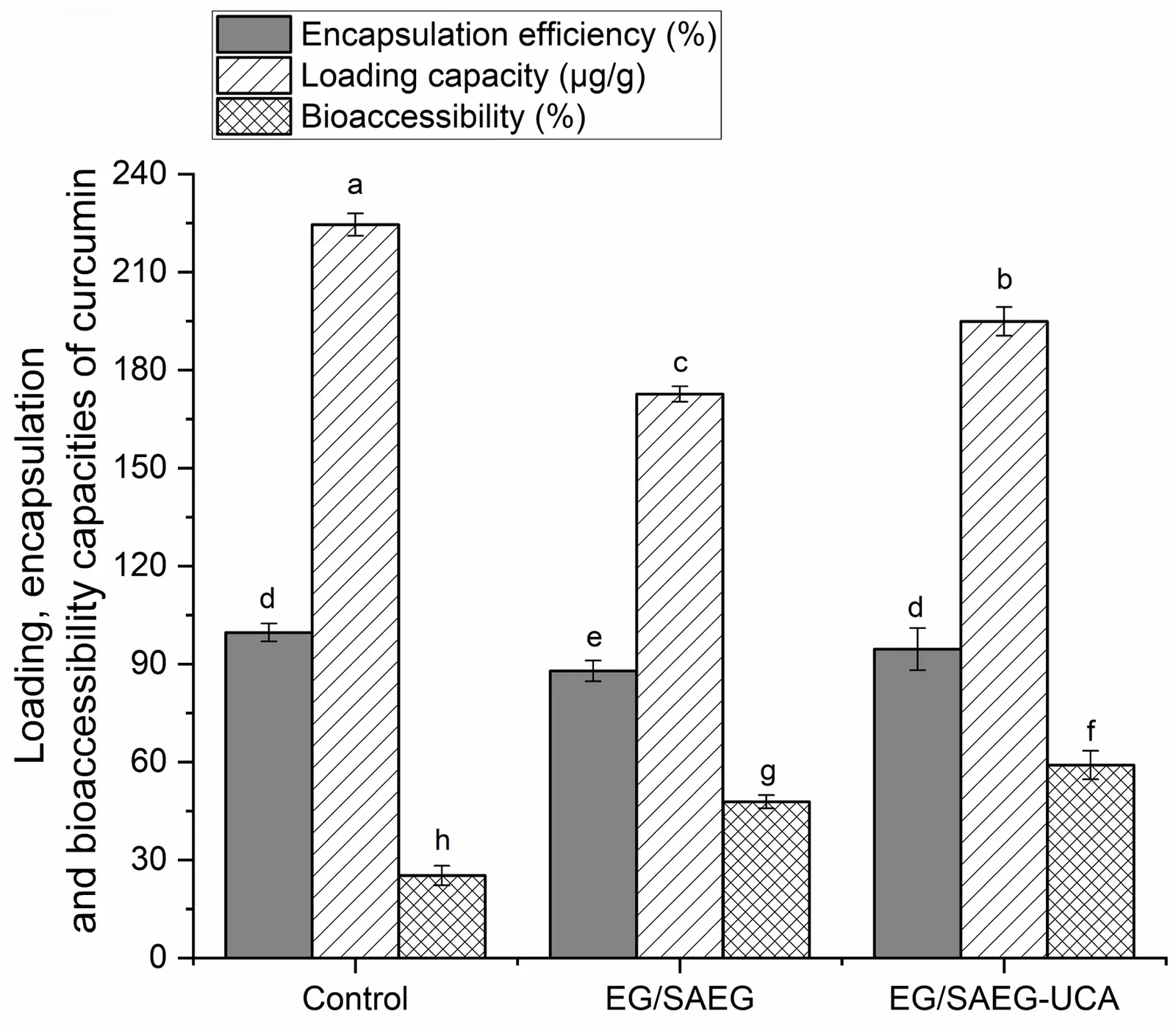
The encapsulation capacity, loading ability, and bioaccessibility of curcumin in the control (corn oil) and the emulsion gels based on SAEG and SAEG-UCA. Different lowercase letters (a–h) on the bars denote statistically significant differences (*p* < 0.05).

As shown in Figure 9, SAEG- and SAEG-UCA-emulsion gels showed superior curcumin bioaccessibility compared with corn oil (*p* < 0.05). The stability of curcumin was crucial for its bioaccessibility [29]. The stability of curcumin in corn oil can be reduced by pH shifts, food compositions, and other factors in the intestine, resulting in a poor bioaccessibility [11]. By contrast, during gastrointestinal digestion of SAEG, free amino acids and peptides were produced consequently, and those products can surround the oil droplets and prevent oil aggregation [18], promoting contact between oil and lipases and the decomposition of the oils; this results in higher curcumin release rates and bioaccessibility. Furthermore, curcumin in SAEG-UCA-emulsion gels exhibited higher bioaccessibility than that in SAEG-emulsion gels (*p* < 0.05). Ultrasonication and chlorogenic acid grafting enhanced the antioxidant stability (Figure 7) and photostability (Figure 8) of SAEG-emulsion gels, thereby promoting the adsorption of curcumin in the intestine. Farooq et al. [13] found that the emulsion gels based on camellia oil and gum Arabic improved the bioaccessibility of curcumin.

Moreover, the loading capacity (172.57 and 194.74 μg/g) and encapsulation efficiency (87.91% and 94.52%) of SAEG- and SAEG-UCA-emulsion gels were equal to those of the emulsion gels stabilized using egg white and soybean proteins [4,10], indicating their potential as curcumin delivery systems. Compared with corn oil, the stable gel network structure of SAEG- and SAEG-UCA-emulsion gels and their high hydrophobic effects and physical protective effects were suitable for encapsulating curcumin. Moreover, SAEG-UCA-emulsion gels exhibited superior encapsulation and loading efficiencies than SAEG-emulsion gels (*p* < 0.05). After ultrasonication and chlorogenic acid grafting, the contents of hydrophobic interactions and disulfide bonds increased (Table 1), which helped enhance the affinity of the gels for curcumin [11]. Additionally, compared to SAEG, SAEG-UCA formed a more compact and stable emulsion gel network structure with higher cohesiveness (Figure 3 and Figure 4, and Table 2), contributing to the higher encapsulation efficiency and loading rate of curcumin [4]. These effects collectively increased the curcumin loading and encapsulation activities of SAEG-emulsion gels.

## 4. Conclusions

The results of this study demonstrate that ultrasonication and chlorogenic acid conjugation improved the emulsifying capacity and emulsion stability of SAEG by enhancing its surface hydrophobicity, interface sorption ability, and solubility, and increasing the zeta potential within the SAEG-emulsion (*p* < 0.05). Moreover, ultrasonication and chlorogenic acid conjugation increased the intermolecular forces (disulfide bonds, hydrophobic effects, and hydrogen bonds), crystallinity, bound water content, and viscosity of the SAEG-emulsion gel, thereby improving its textural properties (hardness, springiness, cohesiveness, and gumminess) and enhancing its rheological stability and elasticity. Furthermore, chlorogenic acid conjugation effectively improved the oxidative stability of SAEG-emulsion gels, and improved curcumin’s photostability, encapsulation and loading capacities, and bioaccessibility. However, the individual effects of ultrasonication on the emulsion gel properties of SAEG and more specific mechanisms should be investigated in future research.

## Figures and Tables

**Table 1:** Intermolecular interactions of the emulsion gels based on SAEG and SAEG-UCA at doses of 6–14 g/100 g.

Addition Amount (g/100 g)	Disulfide Bonding	Hydrophobic Interactions	Hydrogen Bonding	Ionic Bonding
Emulsion gels based on SAEG				
6	0.51 ± 0.05 e	0.52 ± 0.03 d	0.09 ± 0.01 d	0.15 ± 0.02 a
8	0.59 ± 0.04 de	0.54 ± 0.04 d	0.13 ± 0.02 c	0.08 ± 0.02 b
10	0.63 ± 0.06 d	0.71 ± 0.01 b	0.15 ± 0.02 c	0.13 ± 0.01 ab
12	0.72 ± 0.04 c	0.68 ± 0.03 b	0.16 ± 0.02 bc	0.09 ± 0.02 b
14	0.74 ± 0.00 bc	0.69 ± 0.04 b	0.20 ± 0.01 b	0.11 ± 0.00 b
Emulsion gels based on SAEG-UCA				
6	0.54 ± 0.03 e	0.52 ± 0.05 d	0.14 ± 0.02 c	0.09 ± 0.01 b
8	0.65 ± 0.01 d	0.63 ± 0.03 c	0.16 ± 0.00 bc	0.11 ± 0.01 b
10	0.79 ± 0.06 b	0.73 ± 0.05 b	0.19 ± 0.00 b	0.14 ± 0.1 a
12	0.85 ± 0.03 a	0.78 ± 0.04 a	0.23 ± 0.00 a	0.09 ± 0.01 b
14	0.83 ± 0.04 a	0.82 ± 0.09 a	0.26 ± 0.00 a	0.18 ± 0.01 a

SAEG, sweet almond expeller globulin; SAEG-UCA, sweet almond expeller globulin modified by ultrasonication and chlorogenic acid conjugation. Different little letters (a–e) in the same column represent significant differences (*p* < 0.05).

**Table 2:** The texture properties of the emulsion gels based on different concentrations of SAEG and SAEG-UCA.

Concentration (g/100 g)	Hardness (g)	Springiness	Cohesiveness	Gumminess	Chewiness (g)	Resilience
Emulsion gel based on SAEG						
6	47.55 ± 3.27 e	0.51 ± 0.03 e	0.39 ± 0.03 e	24.24 ± 1.87 d	16.36 ± 0.46 f	0.11 ± 0.01 a
8	50.77 ± 2.49 de	0.49 ± 0.02 e	0.40 ± 0.03 e	23.35 ± 0.36 d	15.85 ± 1.44 e	0.10 ± 0.01 a
10	60.14 ± 4.33 c	0.67 ± 0.03 c	0.47 ± 0.0 d	29.32 ± 2.25 cd	20.77 ± 0.09 d	0.10 ± 0.00 a
12	64.40 ± 0.58 bc	0.72 ± 0.02 b	0.43 ± 0.05 e	34.57 ± 3.23 c	21.63 ± 0.75 b	0.09 ± 0.00 a
14	69.28 ± 1.62 b	0.70 ± 0.04 b	0.47 ± 0.02 d	35.51 ± 2.33 c	25.38 ± 0.93 c	0.12 ± 0.01 a
Emulsion gel based on SAEG-UCA						
6	53.47 ± 3.27 d	0.60 ± 0.02 cd	0.47 ± 0.01 d	20.62 ± 1.65 e	19.06 ± 1.05 cd	0.12 ± 0.02 a
8	56.07 ± 2.79 cd	0.66 ± 0.04 c	0.56 ± 0.02 c	27.43 ± 1.69 d	21.76 ± 1.67 c	0.11 ± 0.02 a
10	71.11 ± 4.43 b	0.74 ± 0.01 ab	0.62 ± 0.04 b	36.55 ± 3.00 c	26.48 ± 2.39 c	0.09 ± 0.02 a
12	72.56 ± 3.56 b	0.79 ± 0.03 a	0.63 ± 0.03 b	40.49 ± 2.04 b	28.57 ± 3.01 a	0.07 ± 0.01 a
14	78.63 ± 3.48 a	0.77 ± 0.00 a	0.72 ± 0.02 a	45.17 ± 2.68 a	32.35 ± 1.76 b	0.11 ± 0.02 a

Different little letters (a–f) represent significant differences (*p* < 0.05).

## Data Availability

The original contributions presented in the study are included in the article, further inquiries can be directed to the corresponding authors.

## Abbreviations

The following abbreviations are used in this manuscript:

SAEG	Sweet almond expeller globulin
SAEG-UCA	Sweet almond expeller globulin modified by ultrasonication and chlorogenic acid conjugation
EG/SAGE	Emulsion gel based on sweet almond expeller globulin
EG/SAGE-UCA	Emulsion gel based on sweet almond expeller globulin modified by ultrasonication and chlorogenic acid conjugation
SA	0.6 mol/L of sodium chloride solution
SB	1.5 mol/L of urea solution containing 0.6 mol/L of sodium chloride
SC	8 mol/L of urea solution containing 0.6 mol/L of sodium chloride
SD	8 mol/L of urea solution containing 0.6 mol/L of sodium chloride and 0.5 mol/L of β-mercaptoethanol
